# The Development of a European Multidisciplinary Cleft Lip and Palate Registry by the European Reference Network CRANIO: Experiences, Barriers, And Facilitators

**DOI:** 10.1097/SCS.0000000000010314

**Published:** 2024-05-23

**Authors:** Philip A.J. van der Goes, Saranda Ombashi, Victor van Roey, Malin Hakelius, Irene M.J. Mathijssen, Aebele B. Mink van der Molen, Sarah L. Versnel

**Affiliations:** * Department of Plastic, Reconstructive and Hand Surgery, Erasmus Medical Center, Rotterdam; ‡ Department of Pediatric Plastic Surgery, University Medical Center Utrecht, Utrecht, the Netherlands; † Department of Plastic Surgery and Maxillofacial Surgery, Uppsala University Hospital, Uppsala, Sweden

**Keywords:** Cleft palate, Outcome measures, Pediatrics, midRegistry

## Abstract

The European Reference Network for Rare Craniofacial Aanomalies and Ear-Nose-Throat disorders aims to improve care for patients with such afflictions, including cleft lip and palate (CL/P) across Europe. Cleft treatment remains varied throughout European centers, inhibiting meaningful comparison of treatment outcomes. To overcome these issues, a European-wide common CL/P dataset and registry was developed, facilitating standardized treatment endpoints and outcome measures for international comparison and benchmarking of CL/P centers. Questionnaires and semi-structured interviews were used to determine the set-up of the registry. Previous CL/P initiatives were analyzed to create an initial dataset, refined through consensus meetings. In total, 87 cleft specialists working in specialized CL/P centers from 16 European nations participated. Consensus on a common dataset was reached. A “Level 1” dataset, with mandatory clinical and patient-reported outcome measures, and “Level 2” dataset with additional outcome measures. Finally, 2 dashboards were developed for data dissemination. The development of the European CL/P common dataset and registry tackled challenges with resource disparities, variations in specialists within CL/P teams, regulatory differences in patient data usage, patient-reported outcome measures availability in European languages, and use of assessment tools. This study described the successful development of the European Reference Network for Rare Craniofacial Aanomalies and Ear-Nose-Throat disorders CL/P common dataset and registry. This achievement will help improve patient care and outcomes for patients with CL/P in Europe. Furthermore, this study provides useful information for initiatives with similar aims.

The European Reference Network (ERN) is an initiative of the European Commission and is set on creating (virtual) networks for health care providers.^1^ European Reference Networks aims to improve health outcomes for over 30 million Europeans suffering from rare diseases. The ERN initiative consists of over 900 specialized health care units from 300 health care centers in 26 European countries. This large gathering of expertise has led to the design of 24 distinct ERNs working on a wide range of rare diseases.^1^ One of these 24 initiatives is the ERN for Rare Craniofacial Anomalies and Ear-Nose-Throat disorders (ERN CRANIO).

The objective of the ERN CRANIO is to combine disease-specific expertise, knowledge, and resources from across Europe to achieve health care goals that may otherwise be unachievable in a single country. This includes enhancing clinical skills, improved patient access to high-quality expert care, and increased availability of diagnosis-specific information for health care professionals, patients, and their families.

Furthermore, ERN CRANIO aims to reduce health inequalities across Europe by standardizing practices and making high-quality care, information, and resources accessible to health care providers, patients, and their families/caregivers across Europe, regardless of location.^2^

European Reference Network CRANIO is subdivided into 3 primary diagnostic groups or “workstreams.” First, the craniosynostosis and other craniofacial anomalies workstream. Second, the ear, nose, and throat disorders workstream. Finally, the cleft lip, palate (CL/P workstream), and orodental anomalies workstream.

With an incidence of about 1:1000 in live births, a CL/P is the most common congenital craniofacial anomaly worldwide.^3^ Patients with CL/P can experience a wide range of issues, including, but not limited to, speech impediments, orofacial dysfunction, breathing problems, hearing problems, feeding problems, and psychosocial issues.^4–9^ Patients with CL/P require multidisciplinary care starting at birth and often continuing into adulthood.^10^ Patients with CL/P are exposed to intensive treatment and monitoring protocols, including several surgical interventions, orthodontic treatment, speech and language therapy, hearing assessments, and psychological therapy.^10–13^ However, little scientific evidence exists on what the best practice is. Hence, treatment protocols are based on (local) expert consensus. This resulted in the implementation and use of different CL/P treatment protocols by multidisciplinary CL/P teams throughout Europe and within member states.^14–16^ The Eurocleft study reported that for unilateral CL/P alone, 194 treatment protocols exist within Europe.^17^

Although the overall objectives of these protocols are similar, the protocols differ in the type and timing of outcome measures, technique and timing of surgical procedures, and treatment endpoints.^18^ These differences inhibit the possibility of accurately comparing treatment outcomes of different European cleft centers. Consequently, it is unclear which CL/P protocols result in (un)favorable treatment outcomes. Furthermore, benchmarking between cleft centers remains difficult and is sparsely done. To enable improvement of CL/P treatment outcomes through benchmarking and research, consensus is required between European cleft centers regarding the type and timing of outcome measures and treatment endpoints.

To achieve the objectives set by ERN CRANIO of combining disease-specific expertise, it was important to reach a consensus on a multidisciplinary set of outcome measures relating to CL/P care (common dataset). Moreover, the workstream aimed to use this common dataset set to create a new European-wide, multidisciplinary, CL/P registry. This article describes the steps taken towards consensus on a common dataset. Furthermore, it reports on the development and implementation of the ERN CRANIO CL/P registry of reporting on the experiences, barriers, and facilitators from inception until implementation. Thereby providing valuable information to other initiatives seeking to develop a registry with similar data in a multidisciplinary setting.

## MATERIALS AND METHODS

### Setting up the Project

**FIGURE 1:**
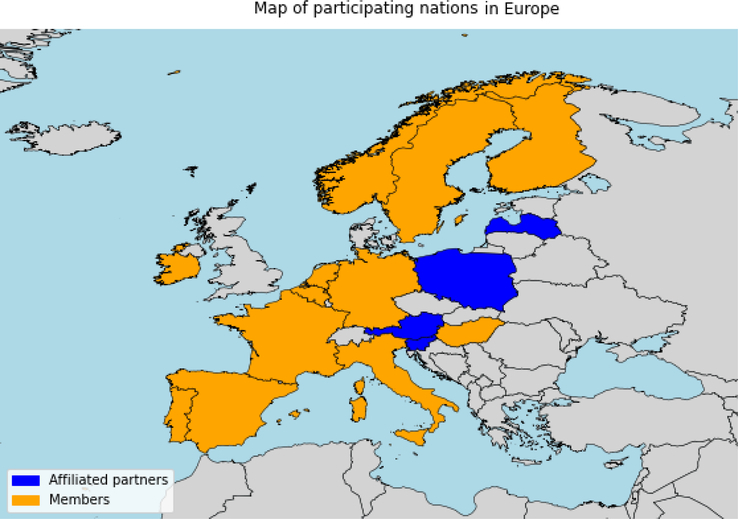
Map of Europe with member states and affiliated partner states in ERN CRANIO CL/P registry. CL/P, cleft lip, palate.

The ERN CRANIO project started in 2017, with the establishment of the ERN coordination office at the Erasmus Medical Center in Rotterdam. Long-term funding for the ERN CRANIO project was procured through the European Commission. Furthermore, a scientific committee and a registry team were set up for each diagnostic group. The CL/P workstream consists of 30 centers from 16 European nations and includes 3 patient representatives, please refer to Figure 1 for a visual overview of distribution throughout Europe.

The CL/P registry team consists of a workstream lead, a project manager, and a dataset researcher. The CL/P registry team had an important role during the first phases of developing the CL/P registry. First, the team was responsible for overseeing the conceptualization and finalization of the common dataset. The content of this common dataset will be explained in detail in the following chapter. Furthermore, with support from the ERN CRANIO coordination office, the team drafted patient information folders, supporting documents such as templates, and specialized manuals for clinicians and patients. Moreover, the registry team is responsible for generating benchmarking reports to enable participating centers to compare treatment outcomes and quality of care, and perform manual data checks on the data entered into the registry. Finally, the registry team supported the ERN CRANIO coordination office by drafting patient consent forms.

For a solid foundation of a common dataset, initiatives such as Scandcleft, Americleft, the NHS standard contract for CL/P, the Swedish CLP registry, and ICHOM set for CL/P were assessed and compiled into a framework.^13,19–24^ This information was used to compile an overview.

A systematic review helped identify key themes for successfully establishing and maintaining a multidisciplinary registry. The outcomes of this systematic review served as a guide for developing the ERN CRANIO CL/P Registry.

Second, experts from cleft centers with experience using medical registries were questioned through semi-structured interviews to gain their opinions and advice regarding a common dataset and a new European-wide, pediatric, and multidisciplinary CL/P registry. The interview framework was created and adjusted iteratively, and the interviews were recorded and transcribed verbatim. Content analyses were performed to identify important experiences, advice, remarks, and opinions.

Lastly, a modified Delphi process was used to gather all information from workstream members regarding facilitators and barriers they anticipated developing a registry. The outcomes of these 3 methods were presented and further discussed at consensus meetings.

## RESULTS

### Conceptualizing the Common Dataset

During the semi-structured interviews and the workstream meetings, it became apparent that 2 main preferences for a common dataset existed within the CL/P workstream. Some centers expressed concern about limited available resources in their health care facilities and requested a common dataset that could be implemented with limited resources. Other centers preferred a more extensive common dataset, allowing for more profound inter-center comparison and possible scientific research in the future.

To facilitate the participation of as many centers as possible within the CL/P workstream without compromising the objectives set by ERN CRANIO, it was decided to use 2 distinct common datasets and thus create a multi-level registry. A “level 1” registry, which focuses on benchmarking with limited resources, was created using the minimal common dataset. Meanwhile, a “level 2” registry was created with the help of cleft centers willing and able to allocate more time and resources toward completing a more extensive common dataset focusing on scientific research. It was important that outcome measures included in the “level 2” common dataset should not exceed what can be expected to fall within the best care for patients with CL/P. For example, additional ionizing imaging could be of interest for research but oversteps the ALARA principle and is thus not considered.

**FIGURE 2:**
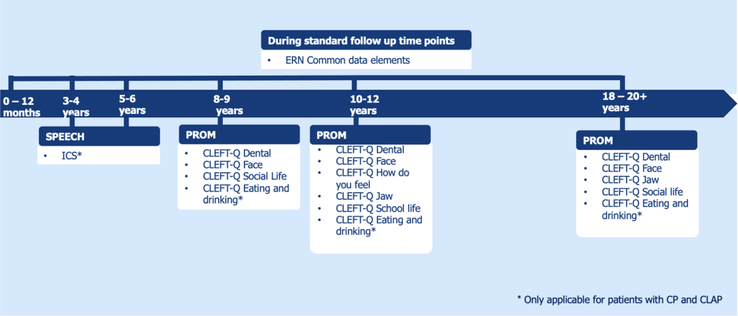
Overview of follow-up protocol for the “level 1” common dataset.

Consensus was reached on the “level 1” common dataset (Fig. 2), containing the ERN common data elements (ECD elements), which are mandatory for all ERN initiatives. The ECD elements were supplemented with the CLEFT-Q scales and the intelligibility in context scal.^24–26^ Both are patient/parent-reported outcome measures (PROMS) and do not require a clinician to complete them. However, these PROMS provide useful data regarding satisfaction of treatment outcomes on different domains such as appearance, function, quality of life, and speech.

After the “level 1” common dataset was composed, the focus of the CL/P workstream shifted towards conceptualizing the “level 2” common dataset. The framework compiled from previous cleft initiatives created at the start of the project was reassessed. Furthermore, members of the CL/P workstream were asked to provide their institutional cleft protocol.

An initial “level 2” common dataset was created. It contained frequently used outcome measures and additional standard assessment times compared to the “level 1” common dataset. The dataset was presented to focus groups arranged separately for each medical specialty to discuss dataset comparability, usability, and implementation feasibility. Please refer to Supplemental Table 1, Supplemental Digital Content 1, http://links.lww.com/SCS/G289 for an overview of the number of participants per specialty.

Supplemental Table 1: Overview of number of participants per specialty for each participating center.

**FIGURE 3:**
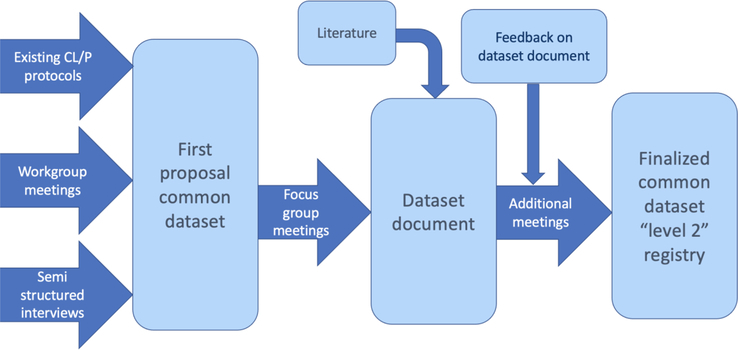
Schematic overview of conceptualization of the “level 2” common dataset.

The outcomes of these discussions, together with the literature consulted, were combined into a dataset document. This dataset document was sent to all participants in the CL/P workstream for feedback, which was collected online and in person during the annual ERN CRANIO congresses. Additional meetings were organized per specialty to reach a final consensus on the common dataset if needed. See Figure 3 for a schematic overview of the conceptualization process of the “level 2” common dataset.

After reaching consensus, the level 2 common dataset contained additional outcome measures, including clinician-reported measures, PROMS, and imaging modalities. An overview of all included outcome measures in the “level 1” and “level 2” datasets can be found in the supplementary files (Supplemental Table A, Supplemental Digital Content 2, http://links.lww.com/SCS/G290). In addition, 4 follow-up protocols have been developed for patients with cleft lip, cleft lip and alveolus, cleft palate, and CL/P. These are also included as supplementary information (Supplemental Figure B-E, Supplemental Digital Content 2, http://links.lww.com/SCS/G290).

### Data Management and Security

Once consensus on a common dataset was reached for both levels, steps towards implementing the registry were taken. A third-party company, “Molgenis,” was hired to help with the technical aspects of the registry. Molgenis is an open-source modular web application used in biobanking, rare disease research, patient registries, and energy research. Furthermore, Molgenis has experience with hosting other registries within the ERN initiative.^27,28^ The CL/P registry team worked closely with Molgenis to design the registry dashboards and the ERN CRANIO CL/P registry website and use their expertise to collect, exchange, and manipulation of large datasets.

As stated previously, the CL/P registry team drafted legal documents, including data-sharing agreements, data ownership and use contracts, and patient consent forms. Acceptance of medical ethical committees in all centers was essential. Therefore, the documents were checked and improved upon by multiple medical ethics committees of participating centers. This was essential to ensure that the participation of centers in the registry would be accepted by other medical ethics committees. Templates for legal documents, created by another ERN initiative “ERICA” were used to help streamline this process.^29^

### Data Entries, Quality Checks, Participation, and Linkage

The CL/P registry was designed to be entirely electronic. This design allows for immediate bulk uploads of data and automated extraction, computation, and data-quality checks, such as plausible ranges, to be implemented. Data can be entered manually on the registry website via bulk uploads that are manually completed or by automating local electronic patient files to generate bulk-upload files according to a standard data template. All patient data in the registry is pseudonymized using the ERDRI.spider tool.^30^

ERDRI.spider pseudonymization tool allows for the linkage of patients within the ERN CRANIO CL/P registry to other ERN registries. This is of particular interest for patients with rare syndromes that suffer from numerous comorbidities on top of CL/P and are therefore included in a different ERN as well (eg, 22q11 syndrome). In addition, this tool will allow patients who have retracted consent to partake in the registry to be de-pseudonymized and removed from the dataset. This is in line with European law, as stated in the General Data Protection Regulation.^31^

In addition, multi-level security is in place to ensure data is always protected. Access for users to contribute data or export data can only be granted via the admin. Furthermore, the Molgenis software allows for immediate data computation and analysis. To tailor the list of questions per specialty, the registry data is organized in a wide format. Consequently, user-friendliness is improved as the data entry process is streamlined and the number of variables for specialists to complete is minimized. Data models are developed using Microsoft Excel, which allows centers to adapt their local data output to fit into the data model.

As shown in Appendix A, outcome measures in both the “level 1” and “level 2” datasets are completed by health care professionals or by patients/parents/caregivers. Clinicians will be responsible for completing all outcome measures except PROMs, which patients willcomplete. Patients or their parents/caregivers will be contacted through their own center to complete the PROMS at the appropriate times. A perceived advantage of PROMs is that patients can complete them from home and thus do not need a physical appointment with their cleft team, thereby expanding opportunities for e-health in cleft care.

All centers participating in the ERN CRANIO CL/P registry must complete the mandatory data entries for the “level 1” registry. Failing to do so may result in the exclusion from the ERN CRANIO initiative. However, the registry team will guide for implementing the registry and may visit local centers to address any issues.

Participation in the voluntary “level 2” registry allows centers to contribute to ERN CRANIO CL/P registry-wide studies.

**FIGURE 4:**
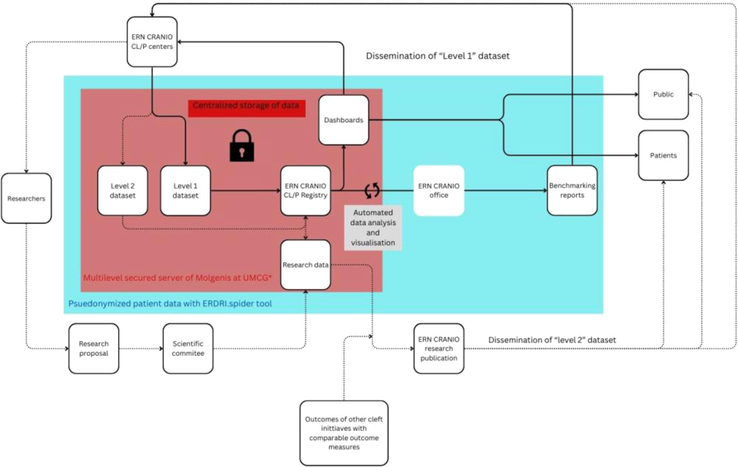
Flowchart of the functioning of ERN CRANIO CL/P datasets and registry; data uploads, storage, analysis, dissemination, and research. CL/P, cleft lip, palate; ERN, European Reference Network.

In Figure 4, the entire process of data collection, uploading, data analysis, and dissemination of information within the ERN CRANIO CL/P common dataset and registry has been visualized.

### Dashboards

At the time of this writing, 2 dashboards have been created. A public dashboard that provides an overview of data that is available to the public. This dashboard provides information on the total number of included patients per diagnosis and locations of participating centers. The clinician dashboard is designed for clinicians from participating centers and reports on the outcomes of the “level 1” dataset. This will provide the participating centers with a fast and profound overview of their treatment outcomes compared to the average of all participating centers without disclosing information to other participating centers. The data presented in this dashboard will not be available to the public. Please refer to the supplemental information for visual examples of both dashboards (Supplemental Appx E-G, Supplemental Digital Content 2, http://links.lww.com/SCS/G290).

## DISCUSSION

### Experiences, Barriers, and Facilitators

The process of conceptualization to implementation of the registry faced several barriers, most of which can be attributed to the inherent differences between individual CL/P centers in Europe and variations in cleft care throughout European nations. These differences occur on many different levels, ranging from institutional or national regulations on patient data usage to the availability of resources, such as differences regarding the number of specialists, specialties included, and experience in multidisciplinary treatment teams, and the presence or absence of a (national) cleft registry.

In addition, there were differences in the usage of assessment tools, creating issues with inter-center compatibility. Language barriers were also evident, with some assessment tools not being available in all CL/P workstream participant languages. Finally, the number of variables included in the dataset and the number of specialties posed a challenge since any changes to the dataset required consensus of the full CL/P workstream. To help the participating centers in the ERN CL/P registry to overcome the barriers mentioned above, the CL/P workstream, the registry team, the scientific committee, and the ERN CRANIO coordination team proposed solutions. To address differences in patient data usage regulations, legal documents were drafted based on already implemented ERN initiatives that adhere to European regulations. Furthermore, specialized manuals were developed to overcome differences regarding assessment tools, such as a manual for orofacial photographs, to guarantee comparable images. These manuals ensure that all participating centers will collect data and use available tools in a similar way, regardless of experience and team size. In addition, the ERN CRANIO coordination team has allocated funding for the translation of assessment tools into all ERN CRANIO languages. This will ensure that language barriers will not prevent any center from participating.

### Future Goals, Improvements, and Research

An important goal of the ERN CL/P registry is to present the collected data to the public, clinicians, patients, and family members/caregivers across different channels.

The next dashboard created will be patient-centered and enable each patient and their health care provider to identify current problems (eg, psychological difficulties needing therapy), and track their own progression throughout treatment.

Another goal is the development of a benchmarking report for each participating center.

In addition, during implementation, centers will receive support from the registry team when they experience difficulty participating.

Besides answering research questions regarding cleft treatment, the cleft registry team will also conduct a careful reassessment of the dataset in the future after initial data collection has been completed.^32^ The dataset reassessment and evaluation of the registry functionality will be performed using the PCDA method.^33^ One of the next steps to further improve the registry is the translation and validation of the CLEFT-Q in all missing ERN CRANIO languages. Successful translation and validation studies have been done for Finnish and Italian in this project. At the time of this writing work is being done on Polish, Slovenian, Hungarian, and Latvian translations and validations.

Furthermore, through standardizing speech assessment throughout the ERN CRANIO CL/P workstream members, we aim to perform a cross-lingual comparison of speech outcomes in patients with CL/P soon.

Finally, requests to use data for scientific studies can be sent to the scientific committee after support of the research proposal by centers in the ERN Cleft workstream. Each center needs to provide approval for the use of their data.

### Limitations

Limitations of this study are no actual patient data has been collected or analyzed within the ERN CRANIO CL/P common dataset and registry. Therefore, we expect that the current dataset to change after data collection, analysis, and evaluation. In addition, numerous years of data collection will be required before meaningful comparison between cleft centers can take place.

## CONCLUSION

The development and implementation of the ERN CRANIO CL/P registry is setting a new standard for cleft care in Europe, and I will facilitate international collaboration and comparison between European CL/P centers. Through the development of a common dataset and CL/P registry, care will improve by enabling benchmarking and scientific research to assess cleft treatment protocols on a previously impossible level. This article provides useful and practical insights for any initiative with similar aspirations by sharing our experiences, barriers, and facilitators during developing a multidisciplinary, international, pediatric (CL/P) registry, from start to implementation.

## Supplementary Material
