# Supplementary material for: The Development of a European Multidisciplinary Cleft Lip and Palate Registry by the European Reference Network CRANIO: Experiences, Barriers, And Facilitators
Source: J Craniofac Surg. 2024 May 23;35(6):1667–72. doi: 10.1097/SCS.0000000000010314 (PMC11346715; doi:10.1097/SCS.0000000000010314)
Supplement: Supplementary file 1 [file scs-35-1667-s001.docx]

| **Country** | **Institute** | **Specialty** | **Prevalence** |
| --- | --- | --- | --- |
| Austria | University Hospital Salzburg | Maxillofacial | 2 |
|  |  | psychologist | 2 |
| Belgium | Antwerp University Hospital | Clinical genetics | 4 |
|  | UZ Gent | Clinical genetics | 1 |
|  | UZ Leuven | ENT | 1 |
| Finland | Helsinki University Hospital | ENT | 2 |
|  |  | Orthodontics | 1 |
|  |  | Plastic surgery | 1 |
| France | Hôpital Universitaire Necker Enfants-Malades | Clinical genetics | 1 |
|  |  | ENT | 4 |
|  |  | Maxillofacial | 1 |
|  | Hopitaux universitaires de Strasbourg | Orthodontics | 1 |
| Germany | Tübingen University Hospital | Maxillofacial | 1 |
|  |  | Pediatrics | 1 |
| Hungary | University of Pécs | Clinical genetics | 2 |
|  |  | Orthodontics | 1 |
|  |  | Plastic surgery | 1 |
| Ireland | Childrens Health Ireland at Temple Street | Clinical genetics | 1 |
|  |  | Plastic surgery | 3 |
|  |  | Psychologist | 1 |
| Italy | AOP University of Padua | ENT | 2 |
|  |  | Psychology | 1 |
|  | San Bortolo Hospital | Maxillofacial | 4 |
|  | San Gerardo Hospital | Maxillofacial | 1 |
|  | Smile House - San Paolo Hospital | ENT | 1 |
|  |  | Maxillofacial | 2 |
|  |  | Orthodontics | 1 |
|  |  | Psychology | 2 |
| Latvia | Riga Stradins University, Cleft Lip and Palate Centre | Maxillofacial | 1 |
| Netherlands | Amsterdam University Medical Center | Plastic surgery | 2 |
|  | Erasmus MC | Clinical genetics | 1 |
|  |  | ENT | 1 |
|  |  | Maxillofacial | 2 |
|  |  | Plastic surgery | 2 |
|  |  | Psychology | 1 |
|  | Radboud MC | Clinical genetics | 1 |
|  |  | Orthodontics | 2 |
|  |  | Psychology | 1 |
|  | UMC Utrecht | Clinical genetics | 2 |
|  |  | ENT | 2 |
|  |  | Maxillofacial | 2 |
|  |  | Orthodontics | 2 |
|  |  | Pediatrics | 1 |
|  |  | Plastic surgery | 2 |
| Norway | Haukeland University Hospital Helse Bergen HF | Clinical genetics | 1 |
|  |  | Plastic surgery | 1 |
|  | Oslo University Hospital | Clinical genetics | 1 |
| Poland | Prof. dr S Popowski Regional Specialised Children's Hospital | Maxillofacial | 3 |
|  |  | Neurosurgeon | 1 |
| Portugal | Hospital de Santa Maria – Centro Hospitalar Lisboa Norte | Plastic surgery | 1 |
|  |  | Psychology | 1 |
| Slovenia | University Medical Centre Ljubljana | Maxillofacial | 3 |
| Spain | Hospital 12 de Octubre | Maxillofacial | 3 |
|  |  | Psychology | 1 |
|  | Hospital de Sant Joan de Déu | Maxillofacial | 1 |
|  | Vall d'Hebron | Clinical genetics | 1 |
|  |  | Maxillofacial | 1 |
| Sweden | Karolinska University Hospital | Clinical genetics | 1 |
|  |  | ENT | 2 |
|  |  | Plastic surgery | 1 |
|  |  | Psychology | 1 |
|  | Sahlgrenska University Hospital | Plastic surgery | 1 |
|  | Uppsala University Hospital | Clinical genetics | 1 |
|  |  | Orthodontics | 1 |
|  |  | Plastic surgery | 1 |
|  |  | Psychology | 1 |
| Europe |  | Patient representatives | 3 |

**Supplemental Table 1:** Overview of number of participants per specialty for each participating center.
