# Supplementary material for: The Development of a European Multidisciplinary Cleft Lip and Palate Registry by the European Reference Network CRANIO: Experiences, Barriers, And Facilitators
Source: J Craniofac Surg. 2024 May 23;35(6):1667–72. doi: 10.1097/SCS.0000000000010314 (PMC11346715; doi:10.1097/SCS.0000000000010314)
Supplement: Supplementary file 2 [file scs-35-1667-s002.docx]

| Classification | Level 1 dataset outcome measures |
| --- | --- |
| ERN Common Data Elements | Date of birth  Sex  Patient status  First contact with specialized center  Age at onset  Diagnosis  Genetic diagnosis  Consent for future research  Consent to reuse data  Biological sample  Link to biobank  Classification of disability |
| PROM | CLEFT-Q |
| Speech | ICS |

# Supplementary information

| Classification | Level 2 dataset outcome measures |
| --- | --- |
| Clinical | Pre-natal checklist  Baseline clinical status  Clinical genetics checklist  ENT checklist  DMFT (decayed, missing, filled teeth)  Overjet + Overbite  Otologic surgery checklist  Dental implant checklist  Orthodontics checklist  Surgical intervention checklist |
| Imaging | 2D-Facial and intraoral photographs  3D-Facial and intraoral photographs  Maxilla and mandible scan/cast  Orthopantomogram + Lateral cephalogram  CBCT |
| Hearing | Hearing level assessment  Audiometry  Tympanometry |
| Speech | VPC  PCC |
| Psychology | SDQ  CATS  PCL5  Social variables questionnaire |
| PROMs | BESAA appearance scale  FACE-Q kids breathing scale |
|  |  |

**Supplemental Table A:** Level 1 and level 2 outcome measures included in the common dataset.


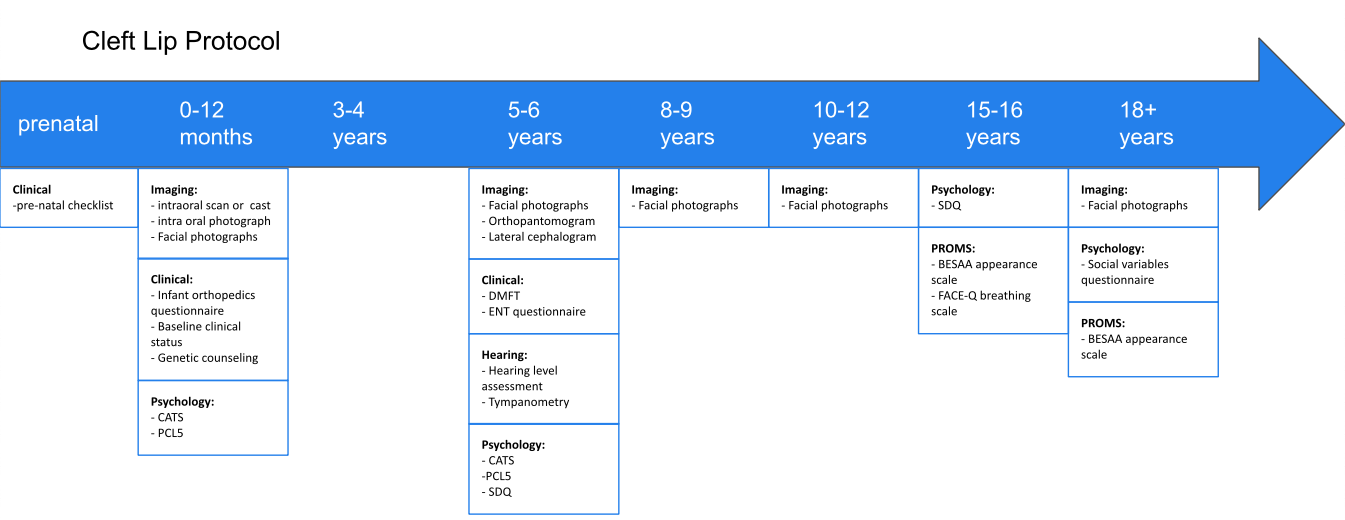


**Supplemental Figure B:** Cleft lip follow-up protocol


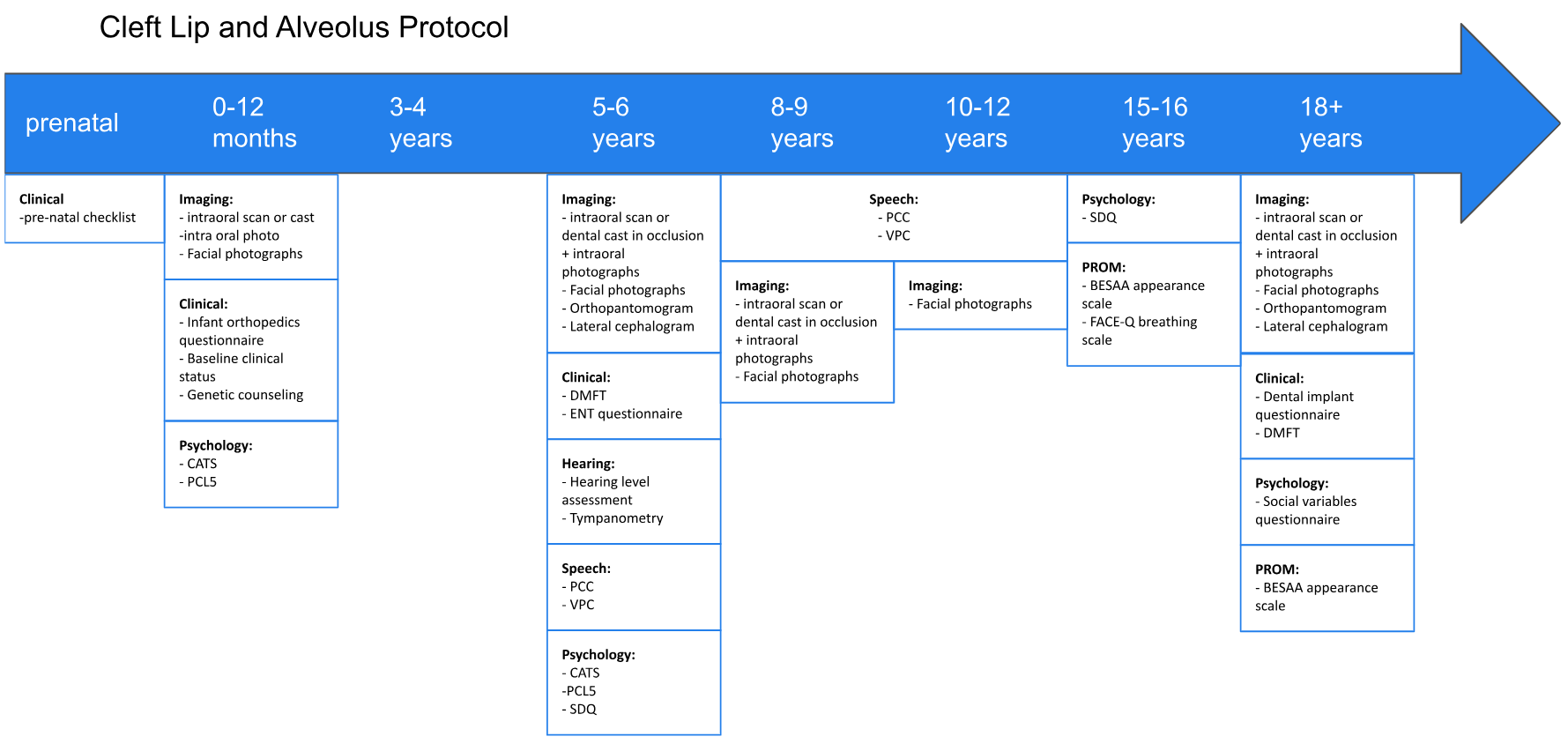


**Supplemental Figure C:** Cleft lip and alveolus follow-up protocol


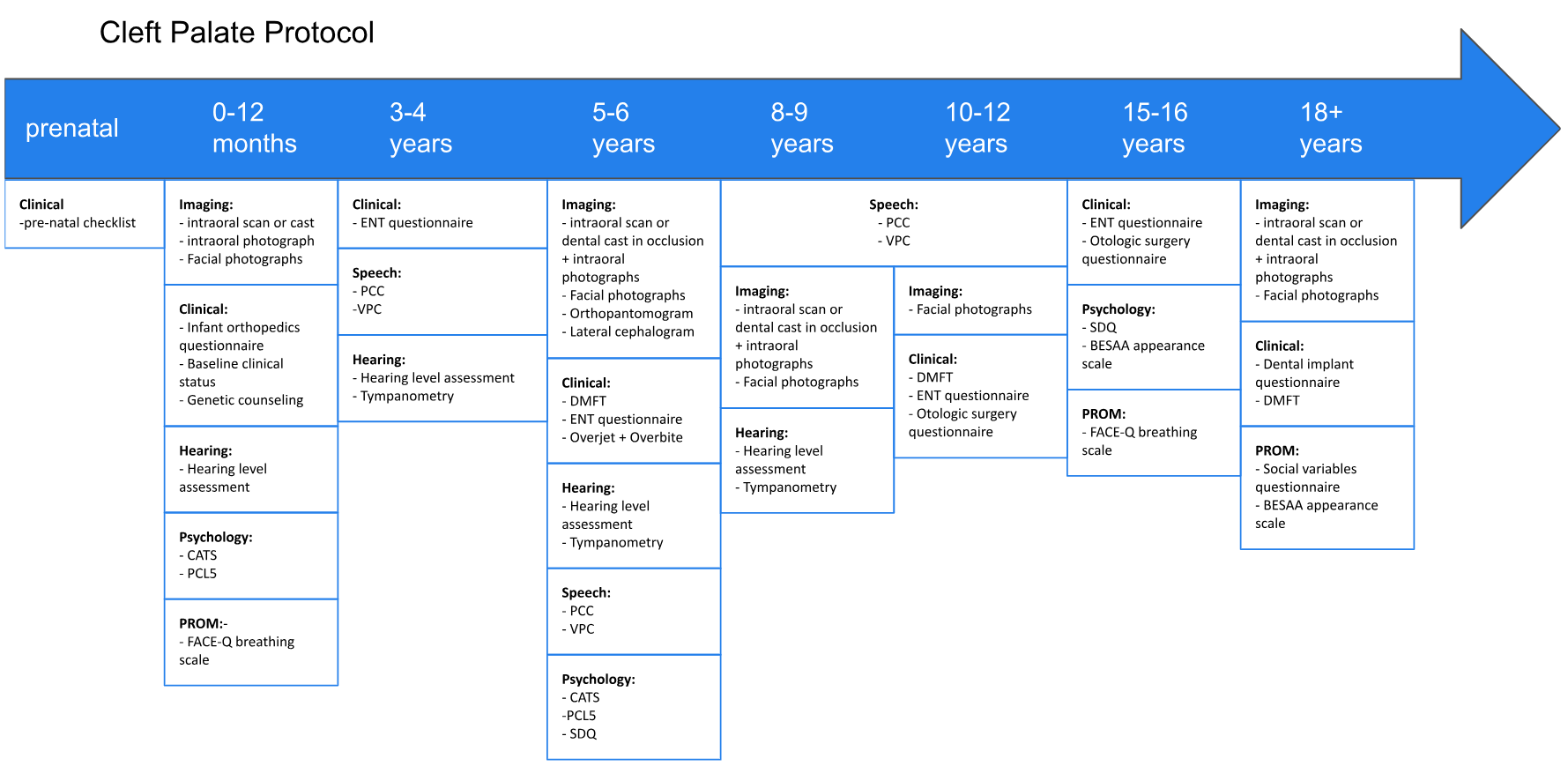


**Supplemental Figure D:** Cleft palate follow-up protocol


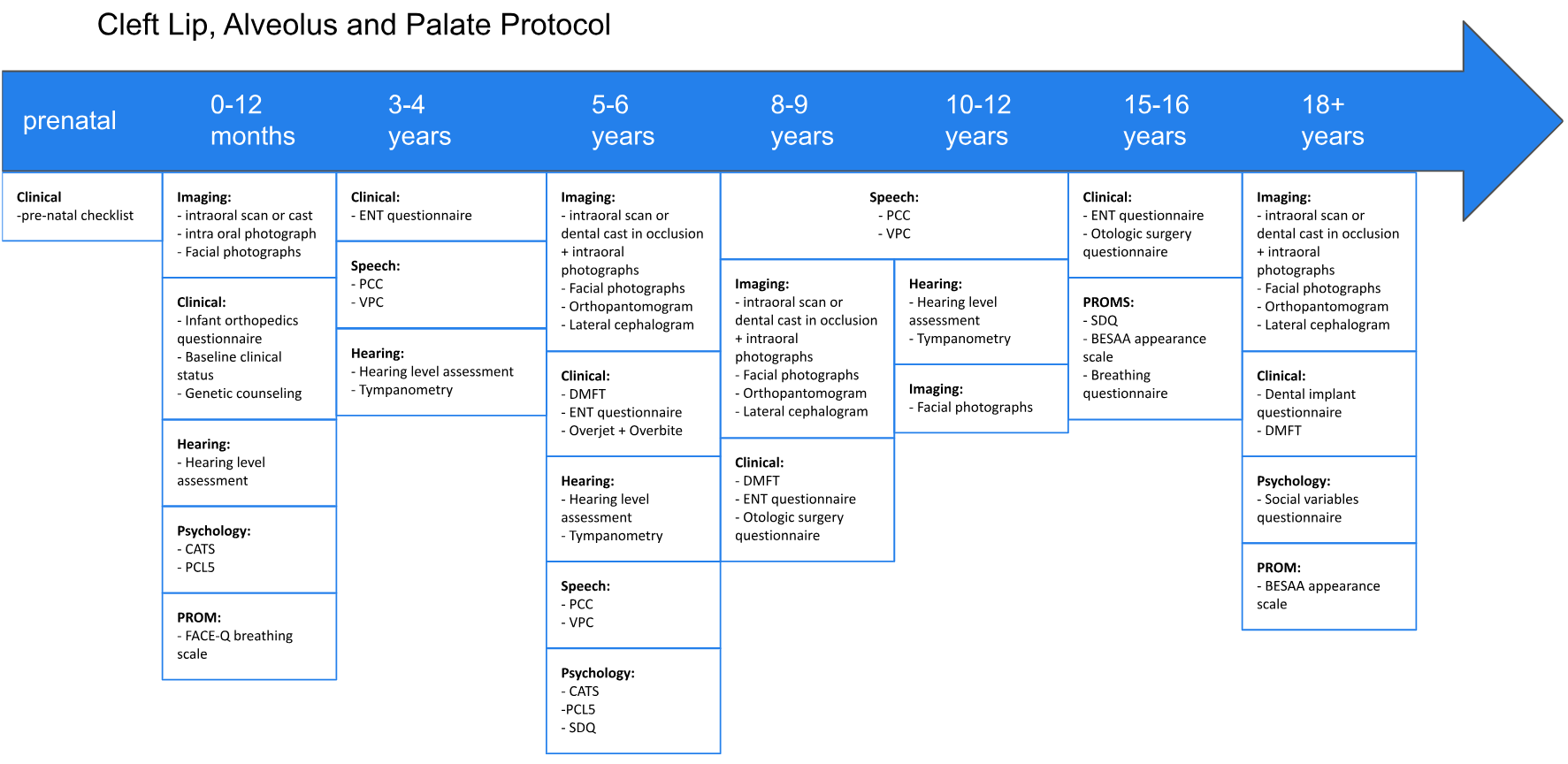


**Supplemental Figure E:** Cleft lip, alveolus and palate follow-up protocol


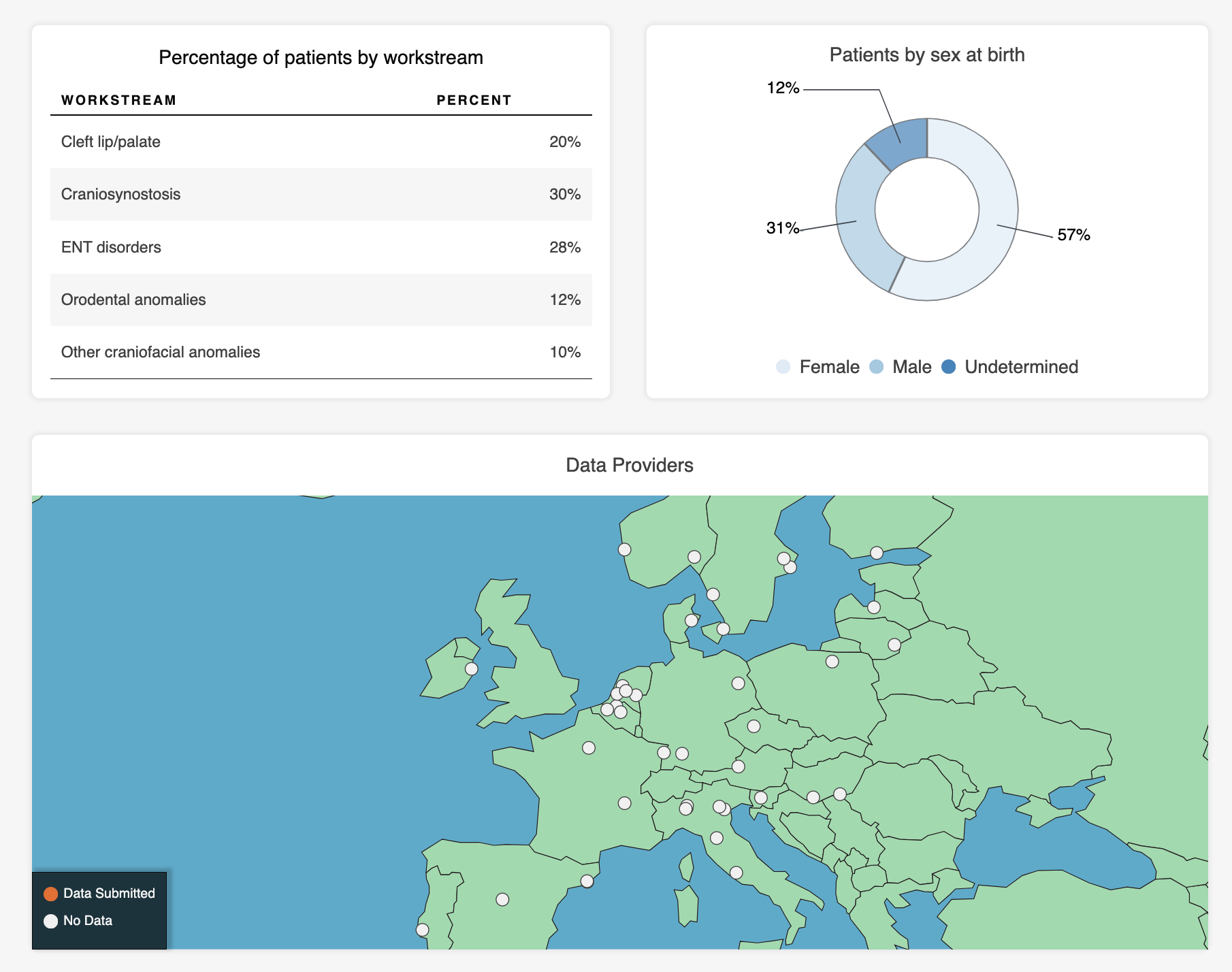


**Supplemental Figure E:** Public dashboard for ERN CRANIO registry, including cleft lip and palate.


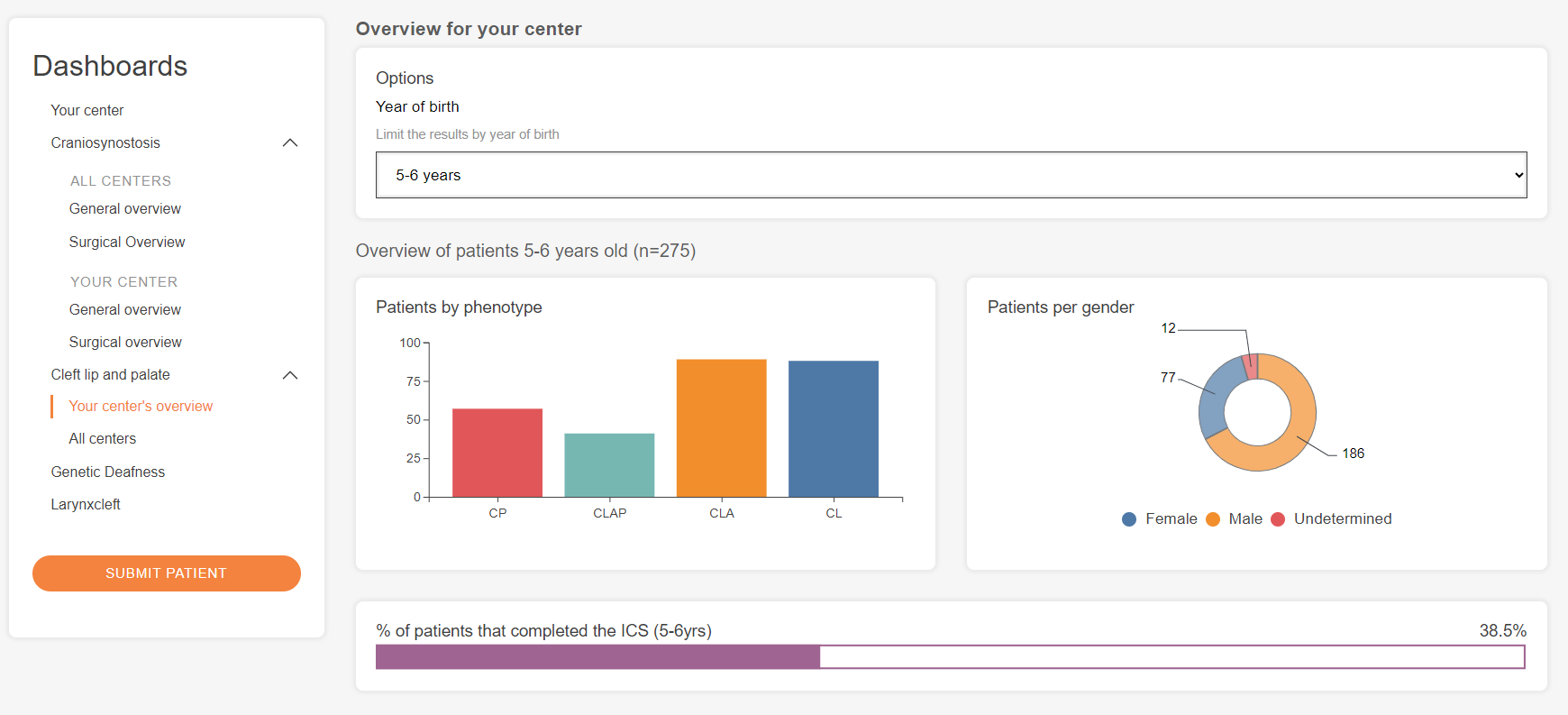


**Supplemental Figure F:** Overview of all CL/P patients in registry


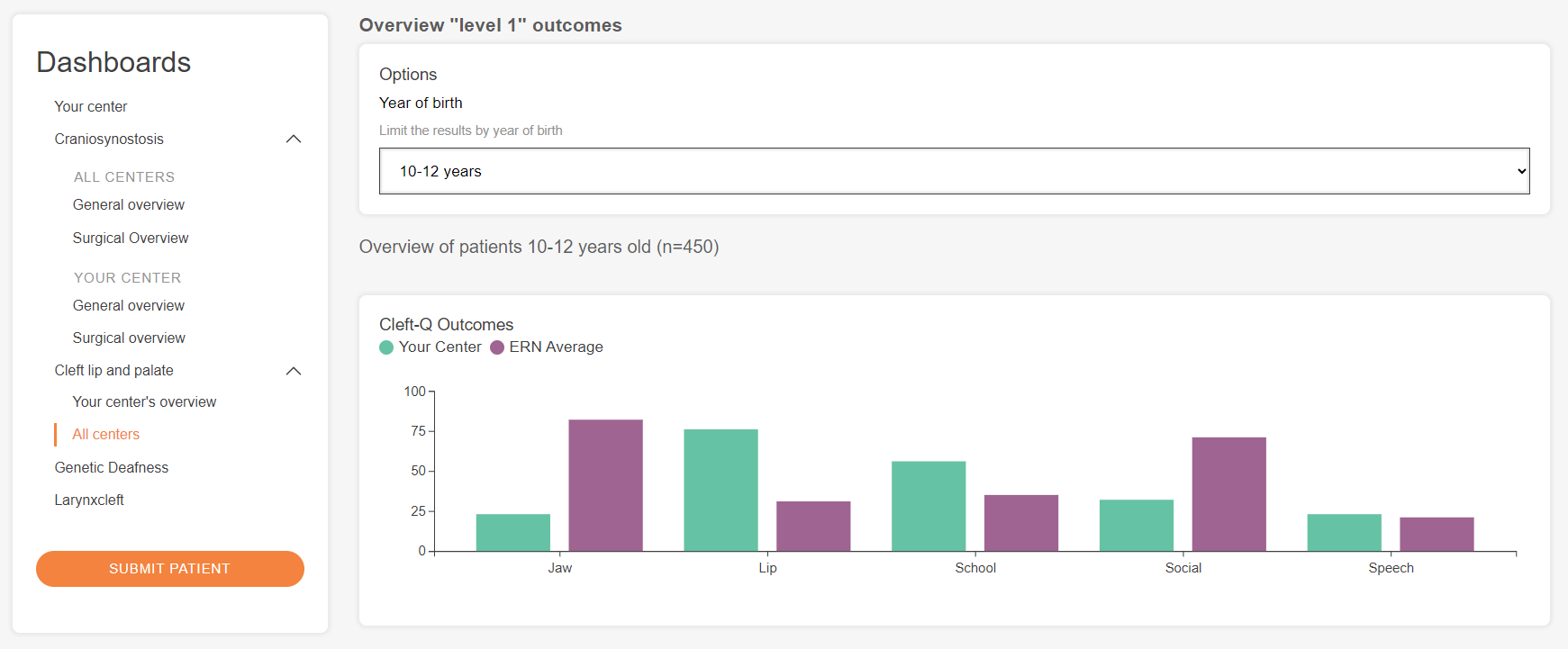


**Supplemental Figure G:** Center specific outcomes for patients with CL/P compared to ERN CRANIO average.
